# Test-retest reliability of Latin American Group for Maturity (GDLAM) protocol in older women

**DOI:** 10.1371/journal.pone.0302134

**Published:** 2024-04-19

**Authors:** Álvaro Huerta Ojeda, Emilio Jofré-Saldía, Jimena Arriagada Molina, Patricia Rojas Quinchavil, María Paz Parada Toledo, Sergio Galdames Maliqueo, María-Mercedes Yeomans-Cabrera, Carlos Jorquera-Aguilera, Frano Giakoni-Ramirez, Maximiliano Bravo

**Affiliations:** 1 Núcleo de Investigación en Salud, Actividad Física y Deporte ISAFYD, Universidad de Las Américas, Viña del Mar, Chile; 2 Escuela de Ciencias de la Actividad Física, el Deporte y la Salud, Universidad de Santiago de Chile USACH, Santiago, Chile; 3 Facultad de Ciencias, Escuela de Nutrición y Dietética, Magíster en Nutrición para la Actividad Física y el Deporte, Universidad Mayor, Santiago, Chile; 4 Centro de Salud Familiar Las Américas, Talca, Chile; 5 Centro de Salud Familiar Faustino González, Talca, Chile; 6 Facultad Ciencias de la Actividad Física y del Deporte, Universidad de Playa Ancha de Ciencias de la Educación, Valparaíso, Chile; 7 Facultad de Salud y Ciencias Sociales, Universidad de Las Américas, Viña del Mar, Chile; 8 Facultad de Ciencias, Escuela de Nutrición y Dietética, Universidad Mayor, Santiago, Chile; 9 Facultad de Educación y Ciencias Sociales, Universidad Andres Bello, Santiago, Chile; 10 Servicio de Medicina Interna, Departamento de Geriatría, Hospital Carlos Van Buren, Valparaíso, Chile; Universidad de Concepción Facultad de Medicina: Universidad de Concepcion Facultad de Medicina, CHILE

## Abstract

Functional autonomy (FA) is a critical factor in determining the quality of life of older adults (OA), especially in the case of older women (OW), as they face a decline in FA in their later years of life. FA should be assessed early, using valid, reliable, and low-cost tests. This study evaluated the test-retest reliability of GDLAM and GDLAM autonomy index (GI) in OW. Thirty-nine OW (71.2 ± 6.50 years) participated in the study. A repeated measures design was used to compare the interday test-retest reliability of the five GDLAM tests (seconds) and the GI (points). The five tests represent activities of daily living, such as dressing or wandering around the house, while the GI provides a weighting of the results of the five tests. The analysis consisted of the intraclass correlation coefficient (ICC), standard error of measurement (SEM), and coefficient of variation (CV). A CV ≤ 10% and an ICC ≥ 0.80 were considered acceptable reliability, whereas a CV ≤ 5% and an ICC ≥ 0.90 were considered high reliability. The outcome of the five tests, represented by the GI, showed high interday test-retest reliability (CV = 6.00% and ICC = 0.91). The results of this study demonstrate that the five tests of the GDLAM protocol and the GI have high interday test-retest reliability and good interday reproducibility. From a practical point of view, the GDLAM protocol allows the assessment of FA of community-dwelling OW, providing background for early diagnosis and, with it, the possibility of developing an individualized physical exercise prescription.

## Introduction

Demographic aging is an emerging and accelerating process [1]. In this regard, in 2018, the United Nations evidenced that people aged 65 and over outnumbered children under the age of five worldwide; likewise, in an estimate for 2050, the number of people aged 80 is projected to reach 426 million, tripling the current figure for that age group [2]. For this reason, the population’s aging and its consequences, the loss of autonomy and independence, are of particular concern to the authorities [3]. Indeed, aging involves changes at the multisystemic level that reduce the capacity for effort and resistance to the physical stress of older adults (OA), decreasing autonomy and quality of life [4].

From a gender perspective, the World Health Organization (WHO) states that women have a longer life expectancy than men [5]. However, despite this longevity, they face a decrease in FA in their later years of life; this situation generates that women are more affected to cope with the demands of activities of daily living, evidenced by a higher prevalence of frailty, a condition that is closely related to both a higher risk of falls and subsequent dependence[6]. Consequently, early assessment of FA in older women (OW) is a priority that contributes to the quality of life of this group of people.

In this context, FA corresponds to a marker of health in OA [7]. FA is defined as the ability to perform activities of daily living or a specific act without the help of others, which is essential to maintaining independence and quality of life [8]. In this sense, FA must be maintained throughout life, especially during the last stage. Consequently, and from the point of view of prevention, it is essential to constantly evaluate this health marker in OA [9]. Early detection of any alteration in FA allows professionals to intervene in a personalized and precise way, helping maintain functionality, delay dependence, and improve people’s quality of life [9].

In practice, different tests and protocols have been used to assess health markers in OA [10–13]. Among them, the application of the Short Physical Performance Battery (SPPB) to determine physical performance parameters [10], the Timed Up and Go (TUG) test to assess fall risk [11] or the Latin American Group for Maturity (GDLAM) protocol to determine FA [12,13]. Despite the number of existing tests and protocols to assess health markers in OA [10,11,14–19], there are still issues to be resolved by science. For example, the SPPB is a valid and reliable test battery for use in geriatric medicine, mainly in institutionalized OA [20]; however, other research claims that the SPPB is not particularly sensitive in OA with higher levels of physical performance, generating a ceiling effect that limits its usefulness [19]. Also, the TUG is a valid test for predicting the risk of falls in OA [21]; however, it has a limited ability to predict the risk of falls in community-dwelling OA [11], as well as lacking different cut-off points to determine frailty in all age ranges [22].

In parallel, the GDLAM protocol was created in Brazil to evaluate the FA of community-dwelling OA [12]. The protocol considers five tests representing daily living activities, while each test’s performance is evaluated in time (seconds). In this way, with the result of the five tests, the Functional Autonomy Index (GI) can be calculated, where a lower score corresponds to a higher FA [12]. The GDLAM protocol has already been used in some regions of South America [12], North America [23] and Spain [24], and qualitative scales based on percentiles have been developed for some specific regions [13,24]. However, to our knowledge, no studies have evaluated the test-retest reliability of GDLAM and GI protocols. For this reason, and to ensure consistent measurements over time, the test-retest reliability of GDLAM in OW should be evaluated, allowing, among other actions: a) informed decision-making by professionals who evaluate and generate protocols to increase FA in OA, b) would facilitate the monitoring of functionality in an accurate and individualized way, c) would allow measuring the effect of interventions and programs in each OA, and d) would avoid the ceiling effect described in other tests [19]. Accordingly, this study aimed to evaluate the test-retest reliability of the GDLAM protocol and the GI in community-dwelling OW. The research hypothesis is that the GDLAM and GI protocols are reliable in interday measures.

## Materials and methods

### Research design

**Fig 1:**
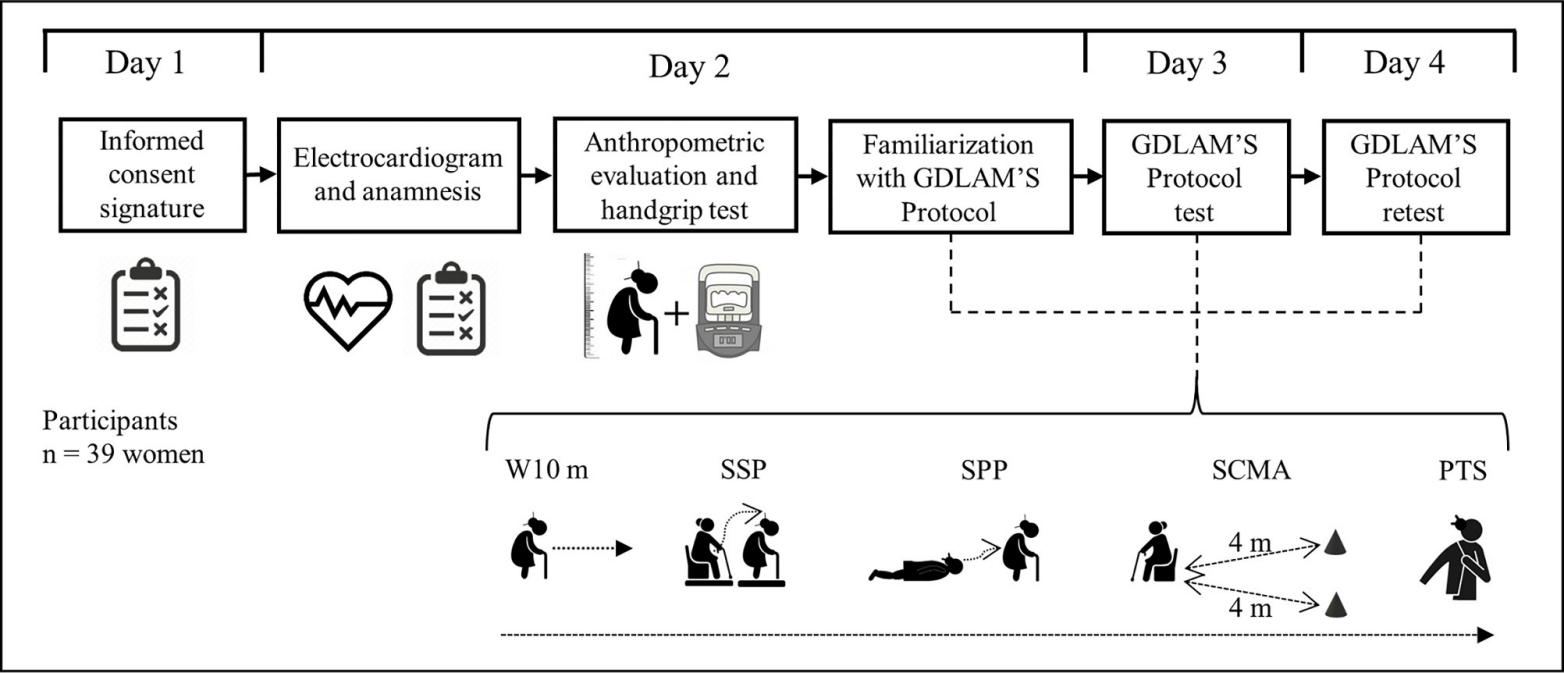
Research design.

A repeated measures design was used to compare the interday test-retest reliability of the five GDLAM and GI tests. All study participants were assessed on four days with a 48-hour interval between assessments. In addition, participants did not exercise between assessment days (Fig 1).

### Participants

Statistical software (G*Power, v3.1.9.7, Heinrich-Heine-Universität, Germany) was used to calculate the sample [25]. The combination of tests used in the statistical software to calculate the sample size was as follows: a) t-test, b) Means: Difference between two dependent means (matched pairs), and c) A priori: Compute required size–given α, power, and effect size. Tests considered two tails, effect size dz = 0.47, *α*-error < 0.05, and a power (1-*β* error) = 0.8. The total sample size was 38 participants.

Thirty-nine OW between 60 and 86 years of age participated voluntarily in this study. According to the background information provided by the participants during the anamnesis, the OW were classified as ’Sedentary’ [26]. The inclusion criteria were the following: being female, being able to move around autonomously and without the aid of a cane or wheelchair (this criterion is due to the staggering sought in the application of the GDLAM protocol), or the assistance of a third party (this criterion is since the GDLAM protocol evaluates FA in self-sufficient persons), having the autonomy to give consent or, if not, being represented by a family member or legal representative. In contrast, the exclusion criteria were the presence of terminal illness, uncontrolled chronic disease, severe cardiovascular conditions, severe pulmonary conditions, fractures in the last three months, neurodegenerative diseases, severe dementia, physical impossibility to performing some of the proposed tests, and refusal to sign the informed consent form. Participant recruitment and evaluations were conducted between October and November 2022. The informed consent form was provided on paper and signed before the assessments started. All participants were informed of the objectives of the study. Initially, 40 women were included in the study. However, one of them could not perform one of the tests autonomously; for this reason, she was excluded. The research and informed consent were approved by the Ethics Committee of the Universidad de Playa Ancha, Chile (registration number: 006–2022). In addition, the study was developed under the ethical standards for exercise and sports sciences [27].

### Anthropometric measurements and handgrip test

For the characterization of the sample, body mass, stature, body mass index (BMI), body fat percentage, and manual gripping strength were evaluated. The maximum hand grip was assessed using a hand-held dynamometer (Smedley® model, Tokyo, Japan) [28]. Before the evaluations, the dynamometer was adjusted to the hand size [29]. The evaluation was performed with the participants seated, with the arm at the side of the body, with the elbow at 90° and the forearm in pronosupination, while the isometric contraction time of the maximal hand grip was 3 to 5 seconds (s) [30,31].

### GDLAM’S protocol

The Latin American Group for Maturity (GDLAM) protocol assessed people’s FA [7,12,13,32]. This protocol considers the application of five functional tests (Fig 1). The unit of measurement for all tests is time in s. A digital stopwatch Casio® HS-70W-1 model (Tokyo, Japan) was used for the time evaluation of the five functional tests. The five functional tests were described by Huerta Ojeda et al. [33]:

#### Walk 10 meters (W10 m)

This test aimed to evaluate the time the participant needed to cover the distance of 10 meters walking without running.

#### Stand up from the sitting position (SSP)

This test aimed to evaluate the strength of the participants’ lower limbs (LL) [34]. This test consists of standing up five consecutive times without assistance or resting the arms on support. The test starts from sitting, while the chair height is 50 centimeters (cm) from the floor. The unit of measurement for this test was timed in s.

#### Stand up from the prone position (SPP)

This test evaluated the participants’ ability to rise from the floor with the arms at the side of the body from the prone position without assistance. The unit of measurement for this test was timed in s.

#### Sit, get up from the chair, and move around the house (SCMA)

This test aimed to evaluate the participants’ agility and balance capacity in daily life situations. With a fixed chair on the floor, with two cones diagonally to the chair (four meters to the back and three meters to the right and left sides, respectively), the participants start the test sitting on the chair. With their feet off the floor, at the ’go’ signal, they stand up, move to the right, turn around the cone, return to the chair, sit down, and remove both feet from the floor. Immediately after that, they perform the same movement to the left. This sequence is repeated twice, emphasizing completing the course as quickly as possible. The unit of measurement for this test was timed in s.

#### To put on and take off a T-shirt (PTS)

This test evaluated the participants’ FA in dressing themselves daily. The test consisted of putting on a T-shirt and taking it off in the shortest possible time. The individual must be standing, with arms at the side of the body and an extra-large (XL) T-shirt held in the dominant hand. On signal, the participant must put on the T-shirt and immediately remove it, returning to the initial position. The unit of measurement for this test was timed in s.

The rest interval between trials was five minutes. Then, the results of the five tests were used to calculate the GI using the following formula [32]:

GI=[(W10m+SSP+SPP+PTS)×2]+SCMA]/4

### Data analysis

Data from the five functional tests of the GDLAM’S protocol, GI, handgrip, and anthropometric parameters were sorted in a spreadsheet designed for the study. Descriptive data are presented as means and standard deviations (SD). Considering there were 39 OW, the normal distribution of the data was confirmed by the Shapiro-Wilk test (*p* > 0.05). Absolute reliability was assessed using the standard error of measurement (SEM) and coefficient of variation (CV) to quantify the systematic error. In contrast, relative reliability was evaluated using the intra-class correlation coefficient (ICC), model 3.1 [35]. All reliability assessments were performed through a customized spreadsheet [36]. Acceptability cutoffs for the ICC were set at ≥ 0.80, whereas ≥ 0.90 was considered high. Acceptable and high thresholds for the CV were set at ≤ 10% and ≤ 5%, respectively [37]. The correlation between the five tests of the GDLAM protocol and the GI was calculated using Pearson’s test [38]. The criteria for interpreting the strength of the *r* coefficients were as follows: trivial (< 0.1), small (0.1−0.3), moderate (0.3−0.5), high (0.5−0.7), very high (0.7−0.9), or practically perfect (> 0.9) [38]. However, Pearson’s correlation cannot detect systematic errors [35]. For this reason, systematic bias was examined through Bland–Altman plots [39]. Although this test is initially recommended to analyze the concordance between two procedures, it has also been suggested and is widely used to complement Pearson’s correlation [35]. All statistical analyses were performed with Prism version 7.00 for Windows® software. The confidence interval for all statistical analyses was 95%, while the significance level for all statistical analyses was *p* < 0.05.

## Results

At the time of the study, the 39 participants were 71.2 ± 6.5 years old, while anthropometric analysis showed a body mass of 67.7 ± 13.0 kg, a stature of 151.2 ± 4.9 cm, and a BMI of 30.2 ± 6.0 kg/m^2^. The anthropometric characteristics are shown in Table 1.

**Table 1:** Characterization of the participants.

	mean ± SD	min	max	95% CI of diff	*p-value* *
**Age (years)**	71.2 ± 6.5	60.0	86.0	69.1 to 73.3	ns
**Body mass (kg)**	67.7 ± 13.0	37.6	98.7	63.5 to 72.0	ns
**Stature (cm)**	151.2 ± 4.9	143.0	161.5	149.6 to 152.8	ns
**BMI (kg/m**^**2**^)	30.2 ± 6.0	17.7	45.6	28.3 to 32.2	ns
**Fat (%)**	33.9 ± 4.0	26.2	41.1	32.6 to 35.4	ns
**Handgrip L (N·kg** ^**-1**^ **)**	1.9 ± 0.7	0.9	3.7	1.7 to 2.2	ns
**Handgrip R (N·kg** ^**-1**^ **)**	1.9 ± 0.6	1.0	3.6	1.7 to 2.1	ns

CI, confidence intervals; kg, kilograms; kg/m^2^, kilograms per meters squared; L, left; m, meters; N, newtons; N·kg^-1^ newton per kilograms of body mass; ns, not significant; R, right

*, Shapiro-Wilk normality test.

The first analysis observed that the mean values of the five functional tests and the IG, both for the test and the retest, are in the "Regular" category [24]. The analysis showed that two tests of the GDLAM protocol presented an acceptable threshold for CV (W10 m = 8.30% and SSP 9.01%), one test presented a high threshold (SCMA = 4.95%), and two tests evidenced a threshold ˃ to 10% between test-retest (SPP = 17.09% and PTS = 11.22%). The same analysis evidenced that one test of the GDLAM protocol presented a high threshold for ICC (SCMA = 0.90), two tests an acceptable threshold (SPP = 0.89 and PTS = 0.86), and two tests showed a < threshold at 0.80 between test-retest (W10 m = 0.74 and SSP = 0.79). Subsequently, when analyzing the GI, a CV equivalent to 6.00% was evidenced, which is considered an acceptable threshold, while the ICC for the GI was 0.91, which is considered a high threshold. This last data allows us to infer that the GDLAM protocol, as a whole, has a low systematic error, which guarantees a high interday reproducibility. The mean values, SD, and 95% confidence intervals (95% CI) are presented in Table 2.

**Table 2:** Test–retest reliability of the GDLAM test.

Variables	Test (mean ± SD)	Retest (mean ± SD)	Δ (95% CI)	SEM (95% CI)	CV (95% CI)	ICC (95% CI)
**W10 m (s)**	8.12 ± 1.23	7.94 ± 1.17	-0.18 -0.49 –-0.12	0.67 0.54–0.86	8.30% 6.79–10.70	0.74 0.55–0.85
**SSP (s)**	13.47 ± 2.74	12.43 ± 2.28	-1.04 -1.57 –-0.50	1.17 0.95–1.50	9.01% 7.37–11.61	0.79 0.64–0.89
**SPP (s)**	6.08 ± 3.01	5.54 ± 2.70	-0.55 -1.00 –-0.09	0.99 0.81–1.28	17.09% 13.97–22.03	0.89 0.79–0.94
**SCMA (s)** *	48.43 ± 7.66	46.02 ± 7.14	-2.40 -3.48 –-1.33	2.34 1.91–3.02	4.95% 4.05–6.39	0.90 0.83–0.95
**PTS (s)**	18.06 ± 5.21	16.67 ± 5.12	-1.40 -2.29 –-0.50	1.95 1.59–2.51	11.22% 9.17–14.45	0.86 0.76–0.93
**GI (points)** *	34.98 ± 6.71	32.79 ± 6.16	-2.18 -3.12 –-1.25	2.03 1.66–2.62	6.00% 4.90–7.73	0.91 0.83–0.95

CI, confidence intervals; CV, coefficient of variation; diff, difference; GDLAM’S Protocol, Latin-American Development to the Maturity Group; GI, GDLAM index of autonomy; ICC, intra-class correlation coefficient; PTS, to put on and take off a T-shirt; SCMA, to sit and get up from the chair and move around the house; s, seconds; SD, standard deviation; SEM, standard error of the mean; SPP, standing up from the prone position; SSP, stand up from sitting position; W10 m, walk 10 meters; Δ, variation delta

*, *p* <0.0001.

**Fig 2:**
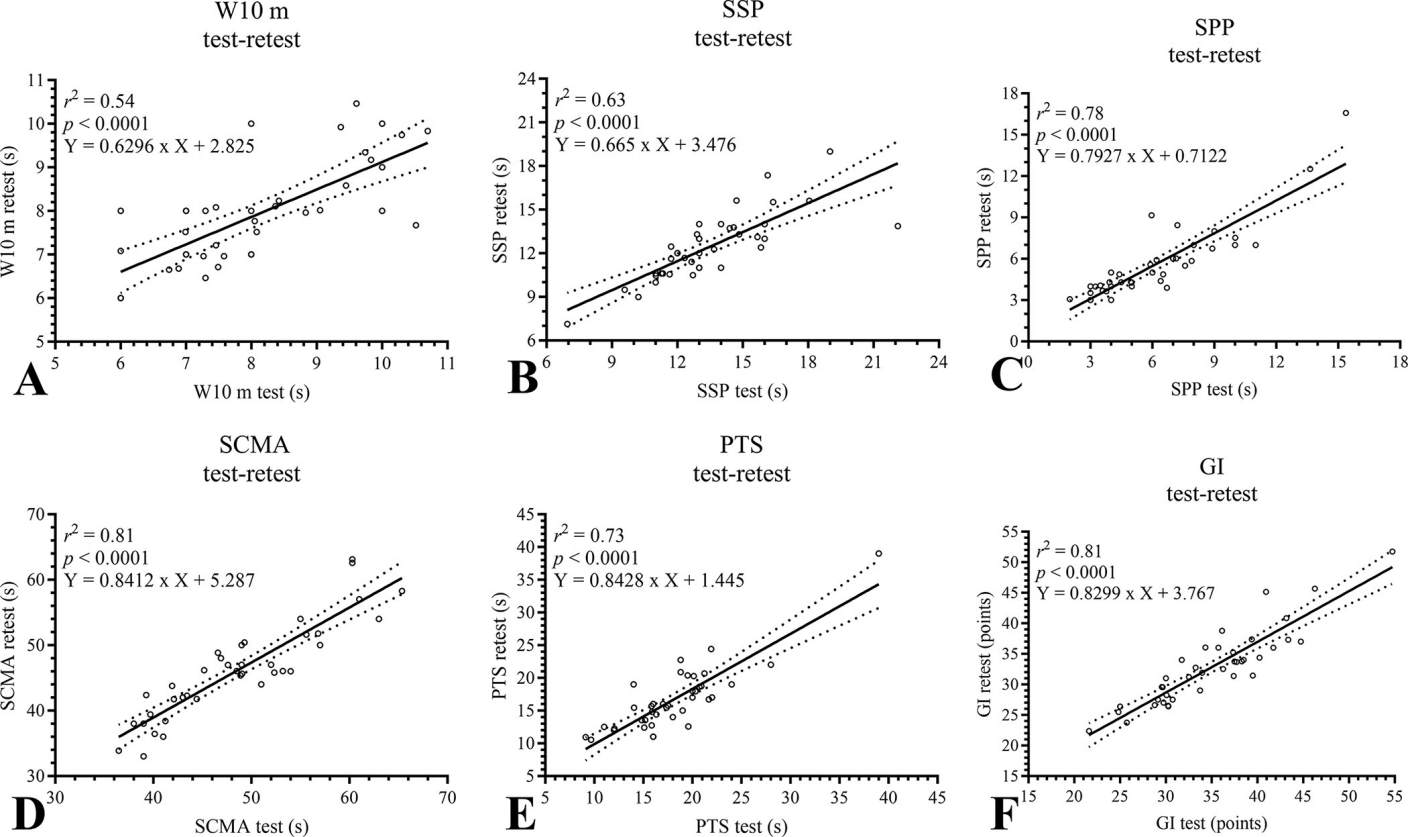
Pearson test and regression line between test-retest of the GDLAM’S protocol. GI, Latin-American Development to the Maturity Group [GDLAM] index of autonomy; PTS, to put on and take off a T-shirt; s, seconds; SCMA, to sit and get up from the chair and move around the house; SPP, standing up from the prone position; SSP, stand up from sitting position; W10 m, walk 10 meters.

The Pearson test results between assessments showed that four tests from the GDLAM protocol exhibited a correlation coefficient (r) ranging from 0.7 to 0.9, considered very high: W10 m *r* = 0.74 (95% CI: 0.56–0.86), SSP *r* = 0.80 (95% CI: 0.65–0.89), SPP *r* = 0.89 (95% CI: 0.79–0.94), and PTS *r* = 0.86 (95% CI: 0.75–0.93). Additionally, one of the tests showed *r* > 0.9, which is considered practically perfect: SCMA *r* = 0.90 (95% CI: 0.82–0.95). Furthermore, when analyzing the test-retest reliability of the autonomy index (GI), a practically perfect *r* > 0.9 was observed: GI r = 0.91 (95% CI: 0.83–0.95). This last data allows us to infer that the GDLAM protocol, as a whole, has a high interday concordance. The graphical representation of Pearson’s test, the respective regression line with *r*^*2*^ values, statistical significance, and the equation are described in Fig 2.

**Fig 3:**
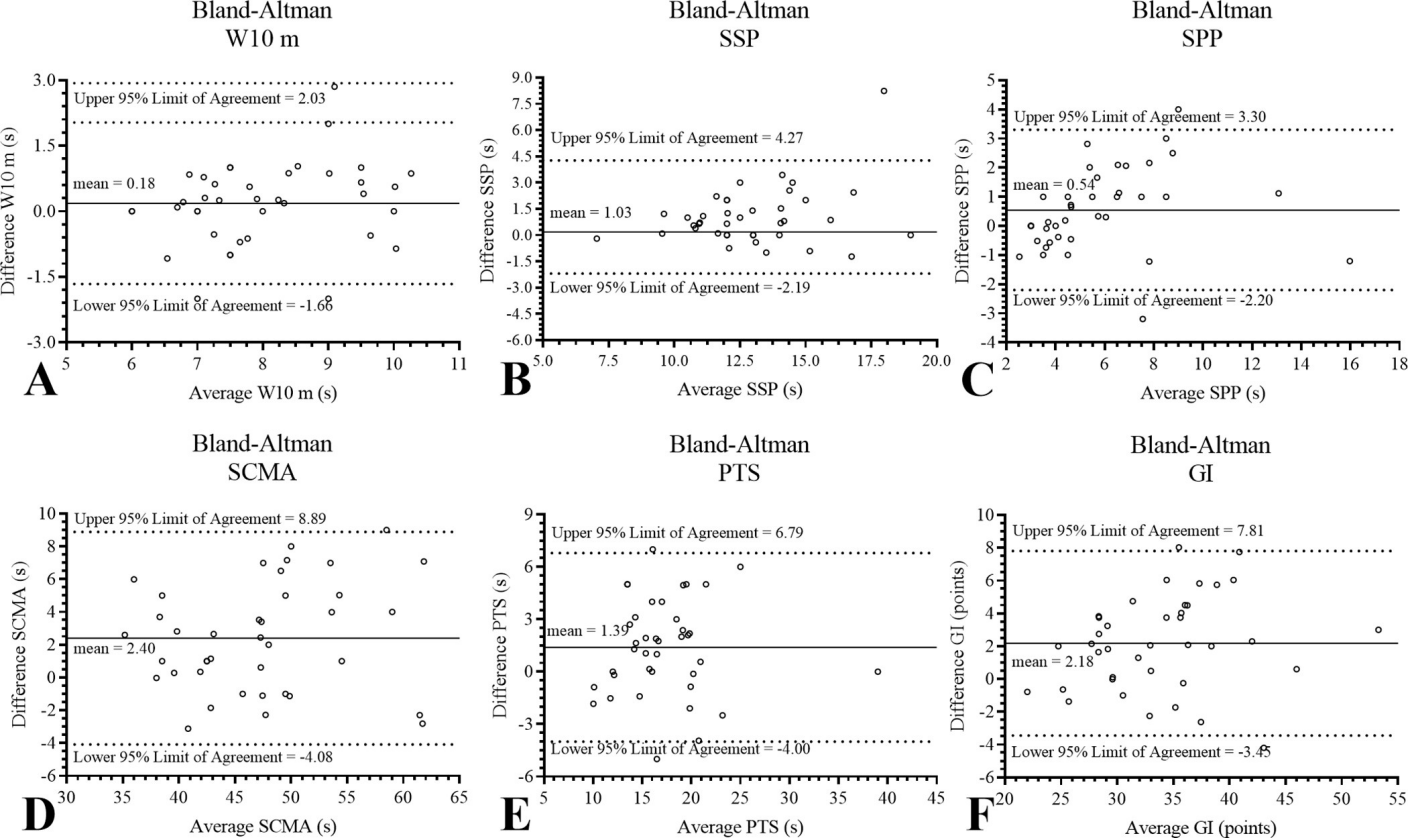
Bland-Altman analysis. The solid line represents the average differences between variables evaluated through a 6-min rowing test. The segmented lines represent 95% of the upper and lower confidence limits. *GI*, *Latin-American Development to the Maturity Group [GDLAM] index of autonomy; PTS*, *to put on and take off a T-shirt; s*, *seconds; SCMA*, *to sit and get up from the chair and move around the house; SPP*, *standing up from the prone position; SSP*, *stand up from sitting position; W10 m*, *walk 10 meters*.

When comparing the mean values and differences of GDLAM and GI protocol between test-retest, the Bland-Altman analysis showed the following results: W10 m a common bias of 0.18 ± 0.94 s, SSP a common bias of 1.03 ± 1.65 s, SPP a common bias of 0.54 ± 1.40 s, SCMA a common bias of 2.40 ± 3.30 s, PTS a common bias of 1.39 ± 2.75 s, GI a common bias of 2.18 ± 2.87 points. Fig 3 shows the graphical representation of the Bland-Altman analysis, with the respective mean difference line and the upper and lower lines of the 95% confidence interval.

## Discussion

This study evaluated the test-retest reliability of the GDLAM and GI protocols in OW. The results of this study showed that both GDLAM and GI protocols have high interday agreement and reproducibility. Consequently, the results suggest that GDLAM and GI protocols are reliable for assessing FA in OW, accepting the research hypothesis.

### Walk 10 meters (W10 m)

This test assesses a person’s linear displacement capacity [40]. Implicitly, the W10 m also assesses dynamic balance and, therefore, can also predict the risk of falls in a manner homologous to other tests that use linear displacement to predict this risk [11]. When the test is used as part of the GDLAM protocol, it is evaluated in s [12]. However, it can also be used independently and quantified in walking speed (m·s^-1^) [41]. From its origins, the W10 m had a CV of less than 5% in test-retest walking speed, demonstrating high interday reproducibility [40]. Other research has recently evaluated this test’s reliability in OA [41,42]. In this regard, Peters et al. [42] reported high inter-test reliability for the W10 m test (ICC = 0.98; 95% CI = 0.96–0.99). Subsequently, Chan and Pin [41] also reported high inter-test reliability for the W10 m test, measured on the 6-min walk test (ICC = 0.91; 95% CI = 0.83–0.95). Concerning the W10 m test evaluated in the present study, our findings showed a CV equivalent to 8.30% (95% CI = 6.79–10.70) and an ICC equivalent to 0.74 (95% CI = 0.55–0.85). These results evidenced acceptable intertest reliability and high interday reproducibility in OW. Despite this high interday reproducibility, the W10 m alone cannot determine FA in OW. In this context, the complement of other tests is needed to represent situations experienced by OW in daily life [12].

### Stand up from the sitting position (SSP)

This test aims to assess lower extremity strength [34]. Transitioning from sitting to standing is considered one of daily life’s most demanding physical activities [43], requiring high energy expenditure [44]. When the test is used as part of the GDLAM protocol, the five repetitions of the SSP are assessed in s [12]. However, it can also be used independently, and each repetition can be quantified in force (kg) or speed of execution (cm·s^-1^) [34]. One of the first validations of this protocol for OA was performed by Guralnik et al. [18]. These investigators reported that the SSP test provides baseline information for the characterization of this age group, specifically of FA [18]. In this regard, a recent study investigated the reliability of the SSP in OA, evidencing an ICC = 0.76 (95% CI = 0.25–0.91) for five repetitions. At the end of the study, the researchers concluded that the SSP test is valid and reliable for assessing lower-limb muscle strength [45]. Concerning the SSP test evaluated in the present study, our results showed a CV equivalent to 9.01% (95% CI = 7.37–11.61) and an ICC equivalent to 0.79 (95% CI = 0.64–0.89), evidencing acceptable reliability and high interday reproducibility in OW. The different SSP or similar protocols used to assess the ability to stand and sit have shown a positive association between lower limb strength and higher FA on OW [33]. In parallel, SSP, as an activity of daily living, has been shown to have a high association with lower extremity functional limitations, self-reported fear of falling, and difficulty climbing stairs among older adults [46]. However, despite the high representativeness of daily living activities and the reliable assessment of lower extremity muscle strength, SSP alone cannot determine FA in OW. In this scenario, as in W10 m, the SSP result must be supplemented with other tests to fully represent the activities of daily living experienced by OW [12].

### Stand up from the prone position (SPP)

This test aims to evaluate the ability to get up from the floor without assistance, starting from the prone position with the arms at the sides of the body. Implicitly, this test evaluates a person’s muscular strength, flexibility, coordination, and balance to perform this complex motor action. When the test is part of the GDLAM protocol, it evaluates in s [12]. However, it can also be used independently and quantified using numerical scales (scores) [47]. In practice, there is evidence that the inability to get up from the floor is relatively common in people with OA living in group housing [48], and, therefore, the ability to get up from the floor unaided is a limiting factor for OA FA. [12]. Concerning the SPP test evaluated in the present study, our results showed a CV equivalent of 17.09% (95% CI = 13.97–22.03) and an ICC equivalent of 0.89 (95% CI = 0.79–0.94), showing acceptable reliability and high interday reproducibility in OW. Independently, the sit-to-stand test has been used as a predictor of mortality, showing that a 1-point increase in score is associated with a 21% reduction in mortality [49]. The SPP test, or its variants, is an easy and safe way to assess an activity of daily living [12]. This test also provides information on the FA of community-dwelling people. It implicitly allows the assessment of other physical capacities of the people evaluated, such as muscle strength and flexibility [49]. However, SPP alone is not capable of determining FA in OW. Therefore, Dantas et al. [12] and Marcos-Pardo et al. [24] decided to include this test in the GDLAM protocol and the GI calculation and thus fully represent the activities of daily living experienced by OW [50].

### Sit, get up from the chair, and move around the house (SCMA)

This test is intended to assess agility and balance in everyday situations involving locomotion, for example, getting in and out of the car, sitting and standing on bus seats, or getting up quickly to answer the doorbell [51]. This test is evaluated in s [12]. To our knowledge, there is no other way to score this test [51]. Before the incorporation of the SCMA test into the GDLAM protocol, the test was validated by Andreotti & Okuma [51], showing an ICC considered high (*r* = 0.99) and a practically perfect correlation coefficient (*r* = 0.96). Concerning the SCMA test evaluated in the present study, our results showed a CV equivalent to 4.95% (95% CI = 4.05–6.39) and an ICC equivalent to 0.90 (95% CI = 0.83–0.95), evidencing high reliability and high interday reproducibility in *OW*. One of the advantages of the SCMA test is its development in several planes and axes of movement, as well as the constant variation of the body’s center of mass; these characteristics make the test very representative of daily life activities [51]. Likewise, during the development of the test, walking speed, agility, balance, and, implicitly, muscular strength can be observed to develop the different transitions of planes and axes of movement. For this reason, the SCMA test is an essential component within the GDLAM protocol, as it allows observing a simulated displacement within a house in activities of daily living [12].

### To put on and take off a T-shirt (PTS)

This test aims to evaluate the ability to dress. In addition, shoulder girdle mobility and coordination between the upper limbs can be observed during the test [12]. Within the GDLAM protocol, this test is evaluated in s, and, to our knowledge, there is no other way to score performance [52]. During the initial validation of the SCMA test, medium-high reliability was recorded (*r* = 0.759, *p* < 0.01) [52]. Concerning the PTS test assessed in the present study, our results showed a CV equivalent to 11.22% (95% CI = 9.17–14.45) and an ICC equivalent to 0.86 (95% CI = 0.76–0.93), evidencing acceptable reliability and high interday reproducibility in *OW*. In this context, the PTS test assesses part of daily living activities, specifically those related to upper extremity function. For this reason, the PTS test is also an essential component within the GDLAM protocol [12].

### GDLAM index of autonomy (GI)

The GI corresponds to a numerical value representing the FA of the persons evaluated through the five tests of the GDLAM protocol. The GI is obtained by relating, through an equation, the five tests of the GDLAM protocol; when obtaining the GI, low numerical values represent a higher FA. Consequently, a lower GI identifies people with greater independence to perform daily tasks [12]. Also, based on the GI, qualitative scales have been proposed to classify the FA of OA in several countries [13,24,32]. Among the advantages of the GDLAM and GI protocol over other tests is the absence of the ceiling effect [19]. This is because the five tests of the GDLAM protocol are measured in seconds. Therefore, there will always be the possibility of improving performance in each of the proposed tests, regardless of the age or sex of the persons evaluated. Another advantage of this protocol is that it is constructed to assess the FA of community-dwelling people (not institutionalized or hospitalized as other tests described in the scientific literature [11]). In this context, cut-off points or qualitative scales developed in countries such as Brazil [13,32] or Spain [24] allow accurate screening of the FA present in individuals from these communities. Regarding the GI calculated by the five tests of the GDLAM protocol of the present study, our results showed a CV equivalent to 6.00% (95% CI = 4.90–7.73) and an ICC equivalent to 0.91 (95% CI = 0.83–0.95), evidencing high reliability and high interday reproducibility in OW and accepting the research hypothesis. In this context, both the GDLAM and GI protocols fulfill the purpose of assessing the FA of community-dwelling OA [12].

### Limitations

Worldwide, OW is a priority group [5,6]. This was the reason for conducting the study only with women. Consequently, the main limitation of this research is the absence of men, so the reliability of the GDLAM and GI protocol in older men is still uncertain. Another limitation is the number of OW evaluated (n = 39). Although there are other reliability investigations with samples similar to the present study [41] or smaller [34], it was not possible to determine the interday reliability in the different OA age ranges proposed in the scientific literature. Despite this, the present study corresponds to the first investigation on the interday reliability of both the GDLAM and GI protocols, marking a milestone in the application and use of this protocol in community-dwelling OA.

## Conclusions

The results of this study demonstrate that the five tests of the GDLAM protocol and the GI have high interday reliability and high interday reproducibility, accepting the research hypothesis. Therefore, this protocol is reliable for assessing FA in community-dwelling older women. Consequently, together with the quantification of FA in community-dwelling older adults, the GDLAM protocol would also allow obtaining a quality-of-life indicator, mainly because a lower GI score is associated with greater independence in performing activities of daily living.

### Perspective

With the determination of the reliability of the GDLAM and GI protocols, the first milestone to investigate the levels and possible changes in FA in community-dwelling OA is met. The following milestones are the association of FA with other variables such as quality of life, environmental pollution, diet, and physical exercise programs, among others.

## Supporting information

S1 Dataset (XLSX)
